# JellyWeb: an interactive information system on Scyphozoa, Cubozoa and Staurozoa

**DOI:** 10.3897/zookeys.554.6745

**Published:** 2016-01-18

**Authors:** Stefano Martellos, Luca Ukosich, Massimo Avian

**Affiliations:** 1 University of Trieste, Department of Life Sciences, via L. Giorgieri 10, I-34127 Trieste, Italy

**Keywords:** Biodiversity informatics, Cnidaria, FRIDA, identification, jellyfish, Medusozoa

## Abstract

Identification of organisms is traditionally based on the use of “classic” identification keys, normally printed on paper. These keys have several drawbacks: they are mainly based on the systematics, requiring identification of orders, families and genera at first; they are written by experts for other experts, in a specific scientific jargon; they have a “frozen” structure (sequence of theses/antitheses); once published, they cannot be changed or updated without printing a new edition. Due to the use of computers, it is now possible to build new digital identification tools, which: 1) can be produced automatically, if the characters are stored in a database; 2) can be freed from the traditional systematics, giving priority to easy-to-observe characters, incl. those usually uncommon to the classical keys, such as ecology and distribution; 3) can be updated in real time once published on-line; 4) can be available on different media, and on mobile devices. An important feature of these new digital tools is their “collaborative” nature. They can be enriched by the contribution of several researchers, which can cooperate while maintaining rights and property of the resources and data they contribute to the system. JellyWeb, the information system on Scyphozoa, Cubozoa and Staurozoa has been developed in Trieste since 2010. The system was created with the aim of – potentially – becoming a starting point for a wide collaborative effort in developing a user-friendly worldwide digital identification system for jellyfishes.

## Introduction

Since the Rio Earth Summit in 1992, access to biodiversity information has become a fundamental task. Biodiversity data are targeted by several efforts of digitalization and aggregation, most of which focus on primary biodiversity data, i.e. natural history collection specimens and field records. Some of these efforts produced wide global networks, e.g. the GBIF (Global Biodiversity Information Facility; Berendsohn et al. 2010, King et al. 2010), which, together with the BioCASE (Biodiversity Collection Access for Europe, Holetschek et al. 2012), is mobilizing ca. 600 millions of records. Primary biodiversity data are mostly used in modeling the distribution of the taxa, and in predicting the effect of climate changes and anthropic pressure on endangered or alien invasive taxa. Taxon related information (nomenclature, auto-ecology, etc.) become the focus of similar large scale efforts only in the last years (Martellos and Attorre 2012, Martellos 2014). The GBIF itself is starting to aggregate checklist data (GBIF 2010), while other efforts are focused on molecular data (Field et al. 2011, Holetschek et al. 2012, Wieczorek et al. 2012), and to ecological information (Fegraus et al. 2005). In the field of hydrobiology, some recent examples can be Fish-SPRICH (Brosse et al. 2013) and Fish-AMAZBOL (Carvajal-Vallejos et al. 2014). In the case of jellyfishes, online resources are however scarce, but some relevant exceptions (e.g., the Jellyfish Dataset Initiative, http://www.bco-dmo.org/dataset/526852).

Digital identification keys are a particular case in the world of biodiversity informatics. Since the development of the DELTA language (Dallwitz 1980), efforts aiming at creating online digital identification keys followed several approaches. The resulting products differ in usability, accessibility, size, etc. (Nimis and Martellos 2009, Hagedorn et al. 2010, Randlane et al. 2010, Martellos and Nimis 2015). With the development of FRIDA (FRiendly IDentificAtion, Martellos 2010), the researchers of the Department of Life Sciences, University of Trieste, aimed at producing a simple but effective instrument for the development of digital identification keys in collaborative efforts. This led to the publication – in the framework of project *Dryades*, and of the EU projects KeyToNature (http://www.keytonature.eu), SiiT (http://ww.siit.eu) and CSMON-LIFE (LIFE13 ENV/IT/842, http://www.csmon-life.eu) – of ca. 600 different digital identification keys for several groups of organisms.

As far as Scyphozoa, Cubozoa and Staurozoa are concerned, there are digital databases hosting taxonomic information, such as WoRMS (World Register of Marine Species, http://www.marinespecies.org/), as well as paper printed keys to genera (as an example, see Cornelius 1997; other keys are listed in Morandini et al., 2005). Few examples of digital resources are available in the web, often limited to specific geographic regions, as the Cubozoan and scyphozoan key of the Carolinian Biogeographic Province (Calder 2009), the key to the Scyphozoa and Cubozoa of the South Atlantic Bight (Calder and King 2008), and, as far as the Mediterranean is concerned, the web site MeteoMeduse (Boero 2013, http://meteomeduse.focus.it/). The latter, however, is an example of citizen science observatory, and does not provide an identification key. To our knowledge, no comprehensive digital identification tools to species of these taxa exist.

By combining taxonomical, ecological, and morphological and anatomical features into an information system, we developed the so called JellyWeb, a simple tool which allow to researchers and laypersons to identify Scyphozoa, Cubozoa and Staurozoa to the species level. This paper presents the results of this effort, available online at the URL http://dryades.units.it/jelly.

## Methods

Data were collected from several sources in literature. The most relevant are Kramp (1961), WoRMS (http://www.marinespecies.org/), the Scyphozoan Wiki (http://thescyphozoan.ucmerced.edu/), and Mills (1999-). Further sources are listed in Balboni 2008, Benci 2008, Sarto 2009, Sola 2009, Coral 2012, Benci 2012, Savonitto 2012, Ukosich 2014. Other paper are under consideration, and will lead to adding to the database other species for several genera, such as *Atolla* (*Atolla russelli*, *Atolla gigantea*, *Atolla chuni*), *Aurelia* (*Aurelia marginalis*), *Chironex* (*Chironex yamaguti*), *Cyanea* (*Cyanea lamarkii*, *Cyanea rosea*, *Cyanea annaskala*, *Cyanea tzetlinii*, and several other species), *Desmonema* (*Desmonema comatum*, *Desmonema scoresbyanna*), *Drymonema* (*Drymonema gorgo*, *Drymonema larsoni*), *Nausithoe* (*Nausithoe marginata*), *Pelagia* (*Pelagia benovici*) *Tripedalia* (*Tripedalia binata*).

The information system is freely available online at the URL http://dryades.units.it/jelly. It organizes data collected in the last five years by the research unit headed by Massimo Avian, at the Dept. of Life Sciences of the University of Trieste. The researchers which contributed to the project agreed on distributing the data under a Creative Commons, share alike, by attribution 3.0 (CC 3.0 by-sa) license.

The software of the information system has been developed in PHP language. The data are stored in a MySQL database. The system is equipped with a multi-entry query interface (Hagedorn et al. 2010), which operates on both a taxonomic database, and a database of nine easily recognizable morphological characters (see below). The multi-entry interface allows complex queries, which can be a first step in the identification of an organism. The multi-entry query system returns lists of taxa, on which the identification process can continue by using a digital identification system. The latter has been developed by using the FRIDA (FRiendly IDentificAtion) package (Martellos 2010). It operates on a morpho-anatomical database, which hosts ca. 200 characters for several infra-generic taxa of Scyphozoa, Cubozoa and Staurozoa (a revision of the content of the database due to recent taxonomic advancements is ongoing). The output of the digital identification system is a digital identification key to the remaining taxa, which can be used by an interactive interface, or printed out as a dichotomous, illustrated key. The whole key can also be exported in a stand-alone version for mobile devices (Nimis et al. 2012).

The query interfaces have been developed according to the results of several usability tests, conducted in the framework of projects KeyToNature and SiiT, as detailed in Martellos and Nimis (2015). The system is under continuous development, following users’ input.

## Results

JellyWeb hosts several information pages and two query system. The home page (http://dryades.units.it/jelly) provides access to several sections: information, describing how the system works; survey area; query (detailed below); checklist, listing all taxa alphabetically by genus and species name, and providing access to their taxon pages; credits.

The query system is made of two parts.

**Figure 1.**
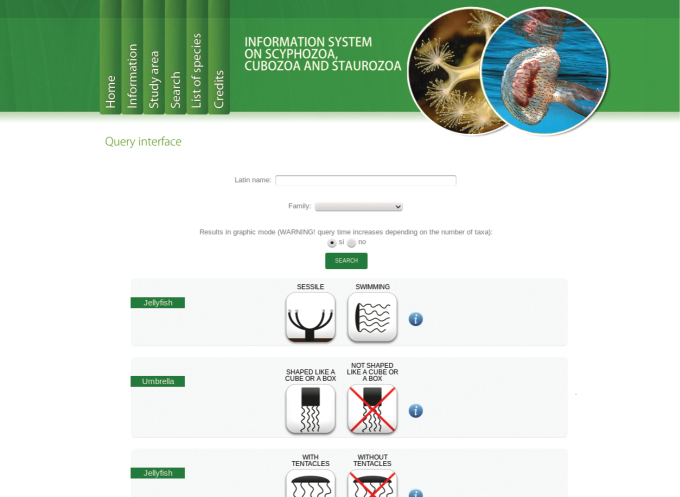
Multi-entry interface. The multi-entry interface allows to combine the states of several morphologic and anatomic characters, together with taxonomic information, to query the database.

**Figure 2.**
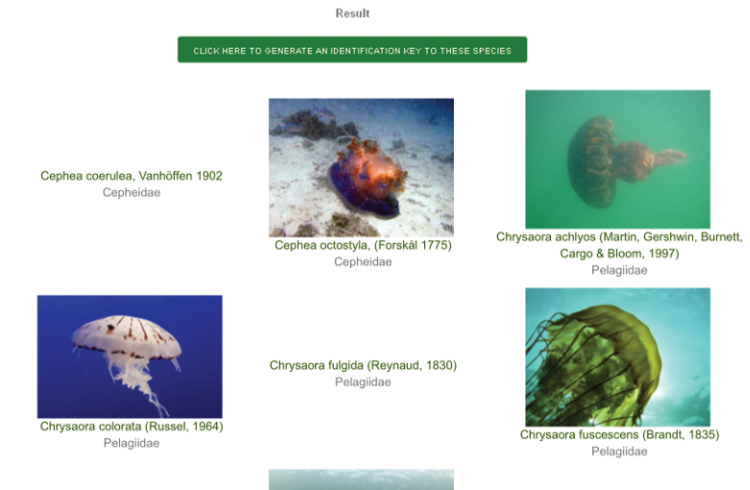
List of taxa. The result of a query made by using the multi-entry interface is an illustrated list of infra-generic taxa.

**Figure 3.**
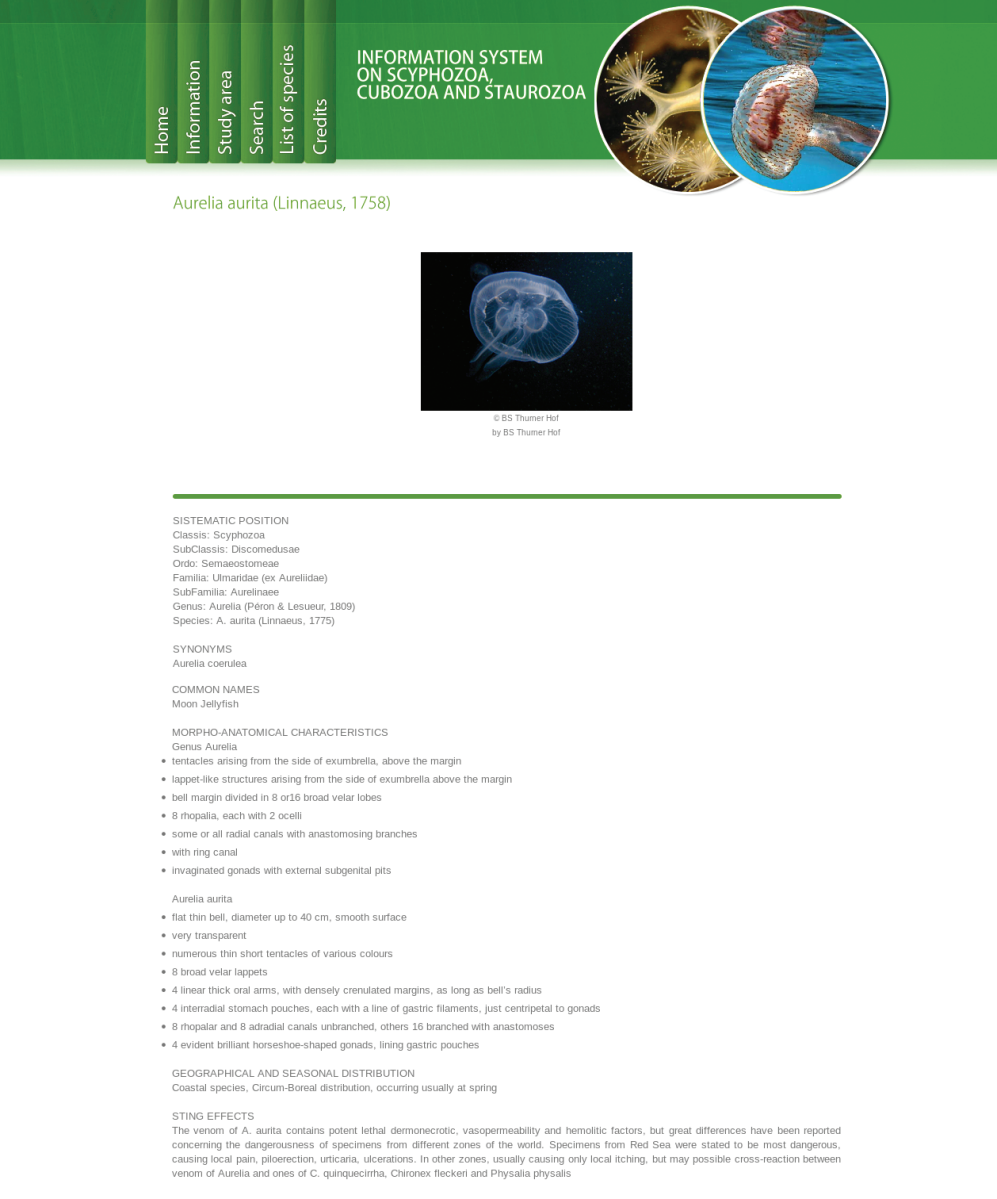
Taxon page. A typical taxon page displays an image, a description, as well as all the other images available in the system, together with credits and metadata. Taxon pages can host a virtually unlimited amount of data, links and media.

**Figure 4.**
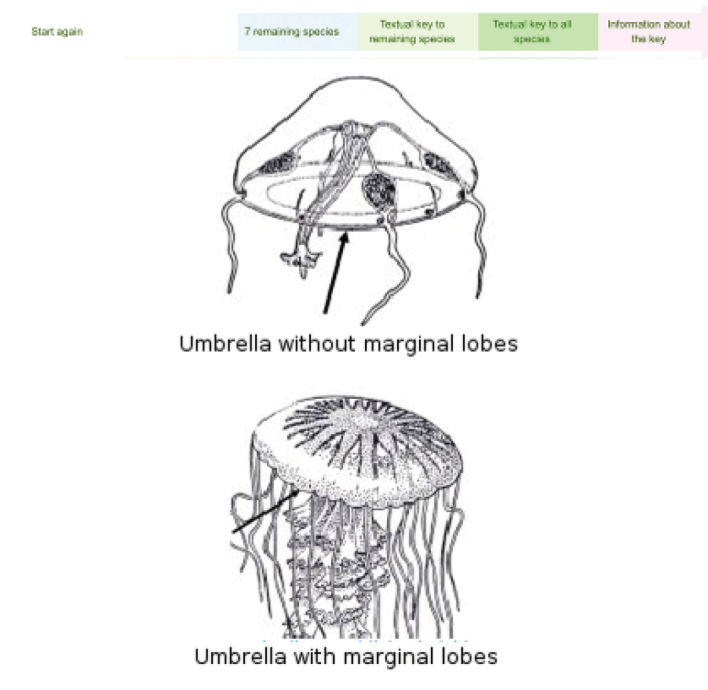
Single entry digital identification key. The digital identification key to remaining taxa is generated from the results of the multy-entry query system. It is used through a single entry interface, and can be printed out as a textual, illustrated dichotomous key as well.

1. Multi-entry interface (Fig. 1). The first interface of the query system provides the users with the opportunity of specifying a set of nine easily observable characters, and/or scientific name and family. The morphological characters are:
  - Jellyfish sessile / swimming;
  - Umbrella shaped like a cube or a box / not shaped like a cube or a box;
  - Tentacles present / absent;
  - Tentacles isolated / grouped in clusters;
  - Umbrella with a coronal groove / without a coronal groove;
  - Umbrella flat / not flat;
  - Oral arms absent / 4 / more than 4;
  - Jellyfish with filaments (oral arm appendages) / without filaments;
  - Jellyfish with scapulae / without scapulae.
  For each character, an information popup window with images and text detailing the most relevat features is accessible by clicking on the question mark button. The result of a query is a list of taxa (Fig. 2). For each taxon an image is displayed (if available, see below). A link provides access to the taxon page (Fig. 3), which displays a description, as well as all the images available in the system, with credits and metadata, and other information (when available). Taxon pages can host a virtually unlimited amount of information and images, and/or provide access to external resources through HTML links.
2. Digital identification key. The results page of the multi-entry interface allows to generate an interactive identification key to remaining taxa. The key can be used through a simple single entry interface (Fig. 4, Hagedorn et al. 2010), or printed out as a textual, illustrated dichotomous key. At each step of the identification process users can list out the remaining taxa, or print an illustrated key. By clicking on a taxon name, the corresponding taxon page is shown (Fig. 3). Each key generated by this system is different from the others, since they contain a different number of infra-generic taxa. Normally, the lower the number of taxa is, the easier the resulting key. A key to all the taxa currently included in our databases can also be generated, and is provided below.

### Dichotomous key to all taxa

This key was automatically generated by the system, and contains all the infra-generic taxa currently stored in our databases at the date October 30, 2015. When a taxon is added to the system the key automatically changes. Hence, the key an user will obtain in the future will be slightly – or completely – different. The keys are not the transposition of an existing paper printed key, but are automatically generated by the system from a database for morphological and anatomical characters by using the package FRIDA (Martellos 2010).

**Table:** 

1	Medusa sessile	**2**
–	Medusa swimming	**34**
2 (1)	Medusa without aboral peduncle	***Lucernariopsis vanhoeffeni* (Browne, 1910)**
–	Medusa with aboral peduncle	**3**
3 (2)	Medusa with sense organs: rhopalioids (anchors)	**4**
–	Medusa without sense organs	**15**
4 (3)	Coronal muscle divided	**5**
–	Coronal muscle unbroken	**10**
5 (4)	Calyx not conical	**6**
–	Calyx conical	**7**
6 (5)	Calyx quadro-pyramidal	***Haliclystus borealis* Uchida, 1933**
–	Calyx pyramidal, octangular	***Haliclystus salpinx* Clark, 1863**
7 (5)	Marginal anchors fairly large, egg-shaped	***Haliclystus stejnegeri* Kishinouye, 1899**
–	Not as above	8
8 (7)	Marginal anchors very large, biscuit-shaped	***Haliclystus antarcticus* Pfeffer, 1889**
–	Not as above	**9**
9 (8)	Marginal anchors kidney-shaped, with a short, cylindric stalk	***Haliclystus auricula* (Rathke, 1806)**
–	Marginal anchors small, oval	***Haliclystus kerguelensis* Vanhöffen, 1908**
10 (4)	Peduncle single-chambered	***Manania hexaradiata* (Broch, 1907)**
–	Peduncle with 4 perradial chambers	**11**
11 (10)	Gonads not united by a transverse circumferential membrane (claustrum) which divide each of the 4 perradial stomach pouches into an outer and an inner space	***Stenoscyphus inabai* (Kishinouye, 1893)**
–	Gonads united by a transverse circumferential membrane (claustrum) which divide each of the 4 perradial stomach pouches into an outer and an inner space	**12**
12 (11)	Calyx as long as wide	***Manania gwilliami* Larson & Fautin, 1989**
–	Calyx longer than wide	**13**
13 (12)	Calyx with dark herringbone pattern	***Manania distincta* (Kishinouye, 1910)**
–	Calyx without dark herringbone pattern	**14**
14 (13)	Arms twice as long as broad	***Halimocyathus platypus* Clark, 1863**
–	Arms short	***Manania handi* Larson & Fautin, 1989**
15 (3)	Peduncle with 4 perradial chambers	**16**
–	Peduncle single-chambered	**22**
16 (15)	Peduncle with muscle in the septa	**17**
–	Peduncle without muscle in the septa	**18**
17 (16)	On each arm about 9 tentacles	***Depastrum cyathiforme* (M. Sars, 1846)**
–	On each arm about 25 tentacles	***Depastromorpha africana* Carlgren, 1935**
18 (16)	Gonads united by a transverse circumferential membrane (claustrum) which divide each of the 4 perradial stomach pouches into an outer and an inner space	**19**
–	Gonads not united by a transverse circumferential membrane (claustrum) which divide each of the 4 perradial stomach pouches into an outer and an inner space	**20**
19 (18)	On each arm 60–80 tentacles	***Craterolophus convolvulus* (Johnston, 1835)**
–	On each arm about 30 tentacles	***Craterolophus macrocystis* von Lendenfeld, 1884**
20 (18)	Arms adradial	***Kishinouyea nagatensis* (Oka, 1897)**
–	Arms interradial	**21**
21 (20)	Arms larger at base than *Sasakiella tsingtaoensis*	***Sasakiella cruciformis* Okubo, 1917**
–	Arms narrower at base than *Sasakiella cruciformis*	***Sasakiella tsingtaoensis* Ling, 1937**
22 (15)	Peduncle without muscle in the septa	***Lucernariopsis campanulata* (Lamouroux, 1815)**
–	Peduncle with muscle in the septa	**23**
23 (22)	Marginal lobes (arms) faintly developed	**24**
–	Marginal lobes (arms) well developed	**26**
24 (23)	Tentacles reduced	***Lipkea stephensoni* Carlgren, 1933**
–	Not as above	**25**
25 (24)	Tentacles not true	***Lipkea ruspoliana* Vogt, 1886**
–	Tentacles rudimentary	***Lipkea sturdzi* (Antipa, 1893)**
26 (23)	Tentacles up to 60 on each arm	**27**
–	Tentacles more than 60 on each arm	**28**
27 (26)	Subumbrellar margin with 4 perradial pigment spots	***Stylocoronella riedli* Salvini-Plawen, 1966**
–	Subumbrellar margin without 4 perradial pigment spots	***Stylocoronella variabilis* Salvini-Plawen, 1987**
28 (26)	Peduncle rudimentary	***Lucernaria australis* Vanhöffen, 1908**
–	Peduncle true	**29**
29 (28)	Peduncle as long or longer than height of calyx	**30**
–	Peduncle shorter than height of calyx	**31**
30 (29)	Tentacles 100–140 on each arm	***Lucernaria quadricornis* O.F.Müller, 1776**
–	Tentacles 700–850 on each arm	***Lucernaria walteri* (Antipa, 1892)**
31 (29)	Tentacles 80 or less on each arm	***Lucernaria infundibulum* Haeckel, 1880**
–	Tentacles more than 80 on each arm	**32**
32 (31)	Peduncle 1/3 as long as height of calyx	***Lucernaria haeckeli* (Antipa, 1892)**
–	Not as above	**33**
33 (32)	Peduncle less than 1/3 of the height of calyx	***Lucernaria bathyphila* Haeckel, 1880**
–	Peduncle about half as long as height of calyx	***Lucernaria sainthilairei* (Redikorzev, 1925)**
34 (1)	Medusa with calix	***Tesserantha connectens***, Haeckel, 1880 – Warning: some authors debate on the validity of swimming Stauromedusae (see Rodriguez et al. 2011)
–	Medusa with umbrella	**35**
35 (34)	Exumbrella divided by a circular and deep coronal groove	**36**
–	Exumbrella not divided by a circular and deep coronal groove	**64**
36 (35)	Tentacles from 4 to 6	**37**
–	Tentacles 8 or more	**42**
37 (36)	Rhopalia 4	**38**
–	Rhopalia 6	**39**
38 (37)	Gonads almost equidistant	***Pericolpa campana* (Haeckel, 1880)**
–	Gonads in 4 pairs	***Pericolpa quadrigata* Haeckel, 1880**
39 (37)	Gonads 6	***Atorella arcturi* Bigelow, 1928**
–	Not as above	**40**
40 (39)	Gonads 8	***Atorella octogonus* Mills, Larson & Young, 1987**
–	Gonads 4	**41**
41 (40)	Gonads sac-like, swollen	***Atorella subglobosa* Vanhöffen, 1902**
–	Gonads leaf-shaped	***Atorella vanhoeffeni* Bigelow, 1909**
42 (36)	Rhopalia up to 6	**43**
–	Rhopalia more than 6	**48**
43 (42)	Rhopalia perradial, 4	**44**
–	Rhopalia interradial, 4	**45**
44 (43)	Coronal muscle divided	***Paraphyllina intermedia* Maas, 1903**
–	Coronal muscle unbroken	***Paraphyllina ransoni* Russel, 1956**
45 (43)	Marginal lappets 32	***Nauphantopsis diomedeae* Fewkes, 1885**
–	Not as above	**46**
46 (45)	Gonads 4	***Periphyllopsis galatheae* Kramp, 1959**
–	Gonads 8	**47**
47 (46)	Marginal lappets 16	***Periphylla periphylla* (Péron & Lesueur, 1809)**
–	Marginal lappets 24	***Periphyllopsis braueri* Vanhöffen, 1902**
48 (42)	Gonads 4 or 4 pairs	**49**
–	Gonads 8	**53**
49 (48)	Stomach pouches break up into numerous ragged-edged branches in the marginal lappets	**50**
–	Stomach pouches simple, radiating	**51**
50 (49)	Subumbrellar protuberances in 2 circles	***Linuche aquila* Mayer 1910**
–	Subumbrellar protuberances in 3 circles	***Linuche unguiculata* (Schwartz, 1788)**
51 (49)	Gonads bean-shaped	***Palephyra indica* Vanhöffen, 1902**
–	Gonads crescent-shaped	**52**
52 (51)	Gonads with horns recurved	***Palephyra antiqua* Haeckel, 1880**
–	Gonads consisting of 3 swellings	***Palephyra pelagica* Haeckel, 1880**
53 (48)	Rhopalia > 8	**54**
–	Rhopalia 8 (Genus ***Nausithoe***. The key refers to free-swimming stages only)	**56**
54 (53)	Gastric ostia with two pigmented spots	***Atolla vanhoeffeni* Russell, 1957**
–	Gastric ostia without pigmented spots	**55**
55 (54)	Species with 20–24 tentacles	***Atolla parva* Russell, 1958**
–	Species with usually 22, sometimes up to 32 tentacles	***Atolla wyvillei* Haeckel, 1880**
56 (53)	Central disk with large pits	**57**
–	Central disk without pits	**58**
57 (56)	Central disk with radiating furrows	***Nausithoe rubra* Vanhöffen, 1902**
–	Central disk without radiating furrows	***Nausithoe atlantica* Broch, 1914**
58 (56)	Gonads very small	***Nausithoe clausi* Vanhöffen, 1892**
–	Not as above	**59**
59 (58)	Gonads of normal dimensions	***Nausithoe albatrossi* (Maas, 1897)**
–	Gonads large	**60**
60 (59)	Central disk not thick nor finely punctured	***Nausithoe globifera* Broch, 1914**
–	Central disk thick, finely punctured	**61**
61 (60)	Central disk with radiating furrows	***Nausithoe challengeri* (Haeckel, 1880)**
–	Central disk without radiating furrows	**62**
62 (61)	Medusa with chocolate brown or carmine gonads and blue gastric cirri	***Nausithoe picta* Agassis & Mayer, 1902**
–	Medusa without chocolate brown or carmine gonads and blue gastric cirri	**63**
63 (62)	Gastric cirri not grouped in clusters	***Nausithoe punctata* (Kölliker, 1853)**
–	Gastric cirri grouped in clusters	***Nausithoe limpida* Hartlaub, 1909**
64 (35)	Opening of the subumbrellar cavity partly closed by an annular diaphragm (velarium)	**65**
–	Opening of the subumbrellar cavity not closed by an annular diaphragm (velarium)	**88**
65 (64)	Tentacles 8 or more	**66**
–	Tentacles from 4 to 6	**76**
66 (65)	Stomach pouches without diverticula	***Tripedalia cystophora* Conant, 1897**
–	Stomach pouches with 8 diverticula	**67**
67 (66)	Gonads not four-leaved	***Chirodectes maculatus* (Cornelius, Fenner & Hore, 2005)**
–	Gonads four-leaved	**68**
68 (67)	Medusa with nematocysts on bell	**69**
–	Medusa without nematocysts on bell	**71**
69 (68)	Each pedalium with more than 4 fingers and tentacles	***Chiropsalmus quadrumanus* Müller, 1859**
–	Each pedalium with 4 or less fingers and tentacles	**70**
70 (69)	Each pedalium with 2 fingers and tentacles	***Chiropsalmus zygonema* Haeckel, 1880**
–	Each pedalium with 3–4 fingers and tentacles	***Chiropsalmus alipes* Gershwin, 2006**
71 (68)	Medusa with mesenteries poorly developed	***Chiropsella bronzie* Gershwin, 2006**
–	Not as above	**72**
72 (71)	Gastric saccules are functioning gonads	***Chironex fleckeri* Southcott, 1956**
–	Gastric saccules are not functioning gonads	**73**
73 (72)	Stomach pouches with 2 branched or feathered saccules	**74**
–	Stomach pouches with 2 unbranched saccules	**75**
74 (73)	Each pedalium with 9–11 fingers and tentacles	***Chirodropus gorilla* Haeckel, 1880**
–	Each pedalium with 21 fingers and tentacles	***Chirodropus palmatus* Haeckel, 1880**
75 (73)	Tentacles and fingers irregularly placed	***Chiropsoides buitendijki* (van der Horst, 1907)**
–	Tentacles and fingers not irregularly placed	***Chiropsoides quadrigatus* (Haeckel, 1880)**
76 (65)	Tentacles branched	***Manokia stiasnyi* Bigelow, 1938**
–	Tentacles simple	**77**
77 (76)	Stomach with weakly developed mesenteries	**78**
–	Not as above	**80**
78 (77)	Sensory niches without well developed covering scale	***Copula sivickisi* Stiasny, 1926**
–	Sensory niches with covering scale above	**79**
79 (78)	Velarial canals 3–4 per octant	***Carybdea marsupialis* (Linnaeus, 1758)**
–	Velarial canals 2 per octant	***Carybdea rastonii* Haacke, 1886**
80 (77)	Stomach without mesenteries	**81**
–	Stomach with well developed mesenteries	**84**
81 (80)	Exumbrella without nematocyst-warts	***Alatina moseri* (Mayer, 190**6)
–	Exumbrella with nematocyst-warts	**82**
82 (81)	Velarial canals 3 per octant	***Alatina rainensis* Gershwin ,2005**
–	Velarial canals 4–5 per octant	**83**
83 (82)	Medusa with 6 eyes per rhopalium	***Alatina madraspartana* Menon, 1930**
–	Medusa with 1 eye per rhopalium	***Alatina tetraptera* (Haeckel, 1880)**
84 (80)	Medusa with phacellae	***Tamoya haplonema* Müller, 1859**
–	Medusa without phacellae	**85**
85 (84)	Velarial canals 1 per octant	***Carukia shinju* Gershwin, 2005**
–	Not as above	**86**
86 (85)	Velarial canals 2 per octant	***Carukia barnesi* Southcott, 1966**
–	Not as above	**87**
87 (86)	Velarial canals 4–5 per octant	***Malo maxima* Gershwin, 2005**
–	Velarial canals more than 5 per octant	***Gerongia rifkinae* Gershwin & Alderslade, 2005**
88 (64)	Medusa with a permanent primary mouth opening in adult specimens	**89**
–	Medusa without a permanent primary mouth opening in adult specimens	**135**
89 (88)	Medusa without tentacles	**90**
–	Medusa with tentacles	**94**
90 (89)	marginal lappets very shallow, or entirely lacking	**91**
–	marginal lappets evident	**92**
91 (90)	Exumbrella transparent white, sometimes with brown nuances on margins	***Deepstaria enigmatica* (Russel, 1967)**
–	Exumbrella reddish-brown, with stomach margin lighter brown	***Deepstaria reticulum* (Lerson, Madin & Harbison, 1988)**
92 (90)	Rhopalia from 24 to more than 50, one in every cleft between the lappets	***Tiburonia granrojo* (Matsumoto, Raskoff & Lindsay, 2003)**
–	Not as above	**93**
93 (92)	Rhopalia 20	***Stygiomedusa gigantea* (Browne, 1910)**
–	Rhopalia 8	***Stellamedusa ventana* (Raskoff & Matsumoto, 2004)**
94 (89)	Medusa with ring-canal	**95**
–	Medusa without ring-canal	**113**
95 (94)	Tentacles arising from umbrella’s margin	**96**
–	Tentacles not arising from umbrella’s margin	**104**
96 (95)	Marginal lappets 48	***Undosa undulata* (Stiasny, 1935)** – Warning: dubious species, some authors suggest it is a juvenile stage of *Discomedusa lobata* Claus 1877
–	Not as above	**97**
97 (96)	Marginal lappets 16	**98**
–	Not as above	**99**
98 (97)	Oral arms broad, egg-shaped	***Ulmaris prototypus* (Haeckel, 1880)**
–	Oral arms narrow and pointed	***Ulmaris snelliusi* (Stiasny, 1935)**
99 (97)	Marginal lappets 32	**100**
–	Marginal lappets 64	**102**
100 (99)	Tentacles 32 or 48	***Discomedusa lobata* (Claus, 1877)**
–	Tentacles 24	**101**
101 (100)	Perradial canals branched	***Discomedusa philippina* (Mayer, 1910)**
–	Perradial canals not branched	***Floresca parthenia* (Haeckel, 1880)**
102 (99)	Tentacles 24	***Parumbrosa polylobata* (Kishinouye, 1910)**
–	Tentacles 16	**103**
103 (102)	Anastomoses absent	***Diplulmaris antarctica* (Maas, 1908)**
–	Anastomoses present	***Diplulmaris malayensis* (Stiasny, 1935)**
104 (95)	Tentacles arising from subumbrella	**105**
–	Tentacles arising from exumbrella	**107**
105 (104)	Gonads 8	***Poralia rufescens* (Vanhöffen, 1902)**
–	Gonads 4	**106**
106 (105)	Rhopalia 8	***Sthenonia albida* (Eschscholtz, 1829)**
–	Rhopalia 16	***Phacellophora camtschatica* (Brandt, 1835)**
107 (104)	Oral arms bifurcated	***Aurosa furcata* (Haeckel, 1880)**
–	Oral arms not bifurcated	**108**
108 (107)	Marginal lappets 16	***Aurelia labiata* (Chamisso & Eysenhardt, 1821)**
–	Marginal lappets 8	**109**
109 (108)	Oral arms short, thick and curved, much folded, extending laterally against subumbrellar surface	***Aurelia limbata* (Brandt, 1835)**
–	Not as above	**110**
110 (109)	Oral arms linear, thick and stiff, with densely crenulated margins, as long as bell’s radius	***Aurelia aurita* (Linnaeus, 1758)**
–	Not as above	**111**
111 (110)	Oral arms narrow and thin, with slightly folded margins only in proximal part	***Aurelia solida* (Browne, 1905)**
–	Oral arms long and broad, curtain-like, with densely crenulated margins	**112**
112 (111)	Adradial canals not branched	***Aurelia maldivensis* (Bigelow, 1904)**
–	Adradial canals branched	***Aurelia colpata* (Brandt, 1838)**
113 (94)	Tentacles arising from the subumbrella at some distance from the margin	**114**
–	Tentacles arising from the exumbrellar margin	**122**
114 (113)	Tentacles not arranged in tufts	***Drymonema dalmatinum* (Haeckel, 1880)**
–	Tentacles arranged in tufts	**115**
115 (114)	Medusa without radial muscolature in the subumbrella	**116**
–	Medusa with radial muscolature in the subumbrella	**118**
116 (115)	Medusa with few broad canals in the lappets	***Desmonema gaudichaudi* (Lesson, 1830)**
–	Medusa with numerous narrow canals in the lappets	**117**
117 (116)	Tentacles not ribbon-like	***Desmonema chierchianum* (Vanhöffen, 1888)**
–	Tentacles ribbon-like	***Desmonema glaciale* (Larson, 1986)**
118 (115)	Rhopalar and tentacular stomach pouches completely separated	**119**
–	Rhopalar and tentacular stomach pouches connected by anastomoses	**120**
119 (118)	Peripheral canals without, or with few anastomoses	***Cyanea capillata* (Linnaeus, 1758)**
–	Peripheral canals with numerous anastomoses	***Cyanea purpurea* (Kishinouye, 1910)**
120 (118)	Peripheral canals with numerous anastomoses	***Cyanea nozakii* (Kishinouye, 1891)**
–	Peripheral canals without, or with few anastomoses	**121**
121 (120)	Radial muscles originating from the outer side of coronal muscle	***Cyanea buitendijki* (Stiasny, 1919)**
–	Radial muscles originating from the middle of coronal muscle	***Cyanea mjobergi* (Stiasny, 1921)**
122 (113)	Stomach pouches 32	**123**
–	Stomach pouches 16	**124**
123 (122)	Subgenital pits heart-shaped	***Sanderia malayensis* (Goette, 1886)**
–	Subgenital pits horseshoe-shaped	***Sanderia pampinosus* (Gershwin & Zeidler, 2008)**
124 (122)	Marginal lappets 16	**125**
–	Not as above	**126**
125 (124)	Nematocyst warts about as long as wide	***Pelagia noctiluca* (Forsskål, 1775)**
–	Nematocyst warts highly protrusive, more long than wide	***Pelagia flaveola* (Eschscholtz, 1829)**
126 (124)	Marginal lappets 48	**127**
–	Marginal lappets 32	**129**
127 (126)	Tentacles all alike	***Chrysaora fulgida* (Reynaud, 1830)**
–	Tentacles different in length	**128**
128 (127)	Tentacles usually 5 per octant, 1 central primary, 2 lateral secondary about half in length, 2 tertiary, between former two types, about 1/4 as long as the median	***Chrysaora lactea* (Eschscholtz, 1829)**
–	Tentacles 5 per octant, 3 primary arising from deep cleft between tentacular lappets and 2 lateral and shorter secondary, arising from subumbrellar side of rhopalar lappets	***Chrysaora quinquecirrha* (Desor, 1848)**
129 (126)	Tentacles 8	***Chrysaora colorata* (Russel, 1964)**
–	Tentacles 24	**130**
130 (129)	Exumbrella yellowish-brown or reddish-yellow with 32-rayed chestnut-brown star	***Chrysaora fusescens* (Brandt, 1835)**
–	Not as above	**131**
131 (130)	Exumbrella reddish-brown or purplish-pink with 16 broad, darker radial bands and numerous light spots	***Chrysaora plocamia* (Lesson, 1830)**
–	Not as above	**132**
132 (131)	Oral arms extremely large with frilly margins, hardly coiled to form a dense mass	***Chrysaora achlyos* (Martin, Gershwin, Burnett, Cargo & Bloom, 1997)**
–	Oral arms linear, with broad frilly margins, more or less coiled around central body	**133**
133 (132)	Exumbrella golden-brown, with darker margins, sometimes with 16-32 lighter radial stripes	***Chrysaora fuscescens* (Brandt, 1835)**
–	Not as above	**134**
134 (133)	Stomach pouches all-alike	***Chrysaora hysoscella* (Linnaeus, 1766)**
–	Stomach pouches unequal, tentacular ones slightly broader proximally and distally than rhopalar ones	***Chrysaora melanaster* (Brandt, 1838)**
135 (88)	Umbrella with papillar knobs	**136**
–	Umbrella without papillar knobs	**138**
136 (135)	Oral arms without filaments	***Lobonemoides sewelli* Rao, 1931**
–	Oral arms with filaments	**137**
137 (136)	Intracircular anastomosing network in communication with the inter-rhopalar canals	***Lobonema smithii* Mayer, 1910**
–	Intracircular anastomosing network not in communication with the inter-rhopalar canals	***Lobonemoides robustus* Stiasny, 1920**
138 (135)	Oral arms dichotomous	**139**
–	Oral arms three-winged	**159**
139 (138)	Medusa with 4 completely separated subgenital cavities	**140**
–	Medusa with 4 not completely separated subgenital cavities	**147**
140 (139)	Oral arms 3/4 the lenght of bell radius	***Cassiopea frondosa* (Pallas, 1774)**
–	Not as above	**141**
141 (140)	Oral arms cylindrical, slender, somewhat longer than bell radius	***Cassiopea ornata* Haeckel, 1880**
–	Not as above	**142**
142 (141)	Oral arms very large, flat, with 6-8 short, wide-spreading main branches	***Cassiopea depressa* Haeckel, 1880**
–	Not as above	**143**
143 (142)	Oral arms 1 1/4 times the lenght of bell radius, triangular in cross-section, aboral surface broad and flat, with 10–15 alternate primary branches	***Cassiopea xamachana* Bigelow, 1892**
–	Not as above	**144**
144 (143)	Oral arms wide, flat, with 4–6 flat, short tree-shaped side branches	***Cassiopea andromeda* (Forskål, 1775)**
–	Not as above	**145**
145 (144)	Oral arms with numerous small lateral branches in their proximal portion	***Cassiopea medusa* Light, 1914**
–	Oral arms cylindrical, 1 1/2 times as long as bell radius, branched tree-like	**146**
146 (145)	Species with numerous large club-shaped vesicles	***Cassiopea mertensi* Brandt, 1838**
–	Species without ribbon-like filaments	***Cassiopea ndrosia* Agassiz & Mayer, 1899**
147 (139)	Oral arms without filaments	**148**
–	Oral arms with filaments	**150**
148 (147)	Exumbrella without a central rised dome	***Marivagia stellata* (Galil & Gershwin, 2010)**
–	Exumbrella with a central rised dome	**149**
149 (148)	More than 1 cupolar warts	***Netrostoma dumokuroa* (Agassiz & Mayer, 1899)**
–	1 cupolar wart	***Netrostoma nuda* (Gershwin & Zeidler, 2008)**
150 (147)	In each octant 3 radial canals	**151**
–	In each octant more than 3 radial canals	**153**
151 (150)	Between the mouths two kinds of appendages	***Netrostoma coerulescens* Maas, 1903**
–	Between the mouths numerous appendages	**152**
152 (151)	Exumbrella with a central rised dome	***Netrostoma setouchianum* (Kishinouye, 1902)**
–	Exumbrella without a central rised dome	***Cephea octostyla* (Forskål, 1775)**
153 (150)	Exumbrella without a central rised dome	***Polyrhiza vesiculosa* (Agassiz, 1862)**
–	Exumbrella with a central rised dome	**154**
154 (153)	Medusa with warts on the central portion of the exumbrella	**155**
–	Medusa without warts on the central portion of the exumbrella	**156**
155 (154)	Radial canals 5–6 per ottante	***Cephea cephea* (Forskål, 1775)**
–	Radial canals 7 per ottante	***Cephea coerulea* Vanhöffen, 1902**
156 (154)	In each octant 4–6 radial canals	***Cotylorhiza erythraea* Stiasny, 1920**
–	Not as above	**157**
157 (156)	In each octant 7–9 radial canals	***Cotylorhiza tuberculata* (Macri, 1778)**
–	In each octant more than 11 radial canals	**158**
158 (157)	Radial canals 11–13 per ottante	***Cotylorhiza ambulacrata* Haeckel, 1880**
–	Radial canals 16–17 per ottante	***Cotylorhiza pacifica* (Mayer, 1915)**
159 (138)	Oral arms triangular	**160**
–	Oral arms not triangular	**162**
160 (159)	Oral arms terminate in a short, oval knob	***Thysanostoma loriferum* (Ehrenberg, 1835)**
–	Not as above	**161**
161 (160)	Oral arms terminate in a long, tapering filament	***Thysanostoma flagellatum* (Haeckel, 1880)**
–	Oral arms without a terminal portion	***Thysanostoma thysanura* Haeckel, 1880**
162 (159)	Oral arms not pyramidal	**163**
–	Oral arms pyramidal	**181**
163 (162)	Oral arms broad	**164**
–	Oral arms of normal width	**169**
164 (163)	Oral arms without filaments	**165**
–	Oral arms with filaments	**166**
165 (164)	Oral arms without terminal clubs	***Lychnorhiza malayensis* Stiasny, 1920**
–	Oral arms with terminal clubs	***Pseudorhiza aurosa* von Lendenfeld, 1882**
166 (164)	Oral arms with terminal clubs	**167**
–	Oral arms without terminal clubs	**168**
167 (166)	Medusa without a single filament at the distal end of one of the oral arms	***Anomalorhiza shawi* Light, 1921**
–	Medusa with a single filament at the distal end of one of the oral arms	***Pseudorhiza haeckeli* Haacke, 1884**
168 (166)	In each octant 8 velar lappets	***Lychnorhiza arubae* Stiasny, 1920**
–	In each octant 4 velar lappets	***Lychnorhiza lucerna* Haeckel, 1880**
169 (163)	Oral arms coalesced throughout their entire length	**170**
–	Oral arms coalesced in proximal portion only	**171**
170 (169)	Velar lappets about 14 in each octant	***Stomolophus meleagris* Agassiz, 1862**
–	Velar lappets about 24 in each octant	***Stomolophus fritillaria* Haeckel, 1880**
171 (169)	Oral arms with filaments	**172**
–	Oral arms without filaments	**175**
172 (171)	Umbrella more than 100 cm wide	***Nemopilema nomurai* (Kishinouye, 1922)**
–	Umbrella less than 100 cm wide	**173**
173 (172)	Velar lappets 14–20 in each octant	***Rhopilema esculentum* Kishinouye, 1891**
–	Velar lappets 8 in each octant	**174**
174 (173)	Exumbrella with sharply conical warts	***Rhopilema hispidum* (Vanhöffen, 1888)**
–	Exumbrella with blunt tuberculation	***Rhopilema nomadica* Galil, Spanier & Ferguson, 1990**
175 (171)	Oral arms with clubs	**176**
–	Oral arms without clubs	**177**
176 (175)	Velar lappets 14–20 in each octant	***Rhopilema rhopalophorum* Haeckel, 1880**
–	Velar lappets 6 in each octant	***Rhopilema verrilli* (Fewkes, 1887)**
177 (175)	Oral arms without terminal clubs	**178**
–	Oral arms with terminal clubs	**179**
178 (177)	Umbrella ca. 150 mm wide; marginal lobes rectagular in shape	***Eupilema scapulare* Haeckel, 1880**
–	Umbrella ca. 400 mm wide; marginal lobes triangular in shape	***Eupilema inexpectata* (Pages, Gili & Bouillon, 1992)**
179 (177)	Proximal portion of oral arms considerably longer than distal portion	***Rhizostoma luteum* (Quoy & Gaimard, 1827)**
–	Proximal portion of oral arms about as long as distal portion	**180**
180 (179)	Taxon present in the Mediterranean and in the Atlantic Ocean	***Rhizostoma pulmo* (Macri, 1778)**
–	Taxon present in the North Sea only	***Rhizostoma octopus* (Macri, 1778)**
181 (162)	Oral arms shorter than usual	**182**
–	Oral arms of normal lenght	**195**
182 (181)	Oral arms without terminal appendages	**183**
–	Oral arms with terminal appendages	**184**
183 (182)	Medusa with rhopalar canals with anastomoses throughout thier length	***Mastigietta palmipes* (Haeckel, 1880)**
–	Medusa with perradial rhopalar canals without anastomoses, interradial canals with anastomoses	***Versuriga anadyomene* (Maas, 1903)**
184 (182)	Intracircular mesh-work of canals never communicating with the rhopalar canals	**185**
–	Intracircular mesh-work of canals usually communicating with the rhopalar canals	**187**
185 (184)	Terminal appendages nearly as long as the oral arms	***Phyllorhiza pacifica* (Light, 1921)**
–	Terminal appendages very long, with distal expansion	**186**
186 (185)	Oral filaments without a triple heart-shaped knob; bell diameter far larger than 25 cm	***Phyllorhiza punctata* (von Lendenfeld, 1884)**
–	Oral filaments with a triple heart-shaped knob; bell of ca. 25 cm of diameter	***Phyllorhiza peronlesueuri* (Goy, 1990)**
187 (184)	Mouth arms twice as long as disk radius	**188**
–	Not as above	**189**
188 (187)	In each octant more than 10 canal-roots	***Mastigias pantherinus* Haeckel, 1880**
–	In each octant up to 10 canal-roots	***Mastigias siderea* Chun, 1896**
189 (187)	Mouth arms shorter than disk radius	**190**
–	Mouth arms long as disk radius	**192**
190 (189)	In each octant more than 10 canal-roots	***Mastigias ocellatus* (Modeer, 1791)**
–	In each octant up to 10 canal-roots	**191**
191 (190)	Vaulted bell, thin at margin but very thick at apex	***Mastigias gracilis* (Vanhöffen, 1888)**
–	Doubtful species, flat and hat-shaped bell, average size unknown	***Mastigias roseus* (Reynaud, 1830)**
192 (189)	In each octant up to 10 canal-roots	**193**
–	In each octant more than 10 canal-roots	**194**
193 (192)	Umbrella not flat	***Mastigias papua* (Lesson, 1830)**
–	Umbrella flat, disk-shaped	***Phyllorhiza luzoni* Mayer, 1915**
194 (192)	Perradial rhopalar canals not bottle-shaped	***Mastigias albipunctatus* Stiasny, 1920**
–	Perradial rhopalar canals bottle-shaped	***Mastigias andersoni* Stiasny, 1926**
195 (181)	Oral arms with filaments	**196**
–	Oral arms without filaments	**199**
196 (195)	Intracircular anastomosing network not in communication with the rhopalar canals	**197**
–	Intracircular anastomosing network in communication with the rhopalar canals	**198**
197 (196)	Distal three-winged portion of oral arms about twice as long as proximal simple portion	***Crambione bartschi* (Mayer, 1910)**
–	Distal three-winged portion of oral arms as long as proximal simple portion	***Crambione mastigophora* Maas, 1903**
198 (196)	Oral arms narrow with short filaments	***Acromitus flagellatus* (Maas, 1903)**
–	Oral arms thick and broad with long filaments	***Acromitus maculosus* Light, 1914**
199 (195)	Oral arms with terminal clubs	**200**
–	Oral arms without terminal clubs	**202**
200 (199)	In each octant 10 velar lappets	***Leptobrachia leptopus* (Chamisso & Eysenhardt, 1821)**
–	Not as above	**201**
201 (200)	In each octant 16 velar lappets	***Crambionella orsini* (Vanhoffen, 1888)**
–	In each octant 12 velar lappets	***Crambionella stuhlmanni* (Chun, 1896)**
202 (199)	Intracircular anastomosing network not in communication with the rhopalar canals	**203**
–	Intracircular anastomosing network in communication with the rhopalar canals	**204**
203 (202)	In each octant 4 cleft velar lappets	***Acromitoides purpurus* (Mayer, 1910)**
–	In each octant at least 5 cleft velar lappets	***Acromitoides stiphropterus* (Schultze, 1897)**
204 (202)	Distal three-winged portion of oral arms 1/6 as long as proximal simple portion	***Catostylus mosaicus* (Quoy & Gaimard, 1824)**
–	Not as above	**205**
205 (204)	Distal three-winged portion of oral arms 6 times as long as proximal simple portion	***Catostylus perezi* Ranson, 1945**
–	Not as above	**206**
206 (205)	Distal three-winged portion of oral arms 5 times as long as proximal simple portion	***Catostylus viridescens* (Chun, 1896)**
–	Not as above	**207**
207 (206)	Distal three-winged portion of oral arms half as long as proximal simple portion	***Catostylus tripterus* (Haeckel, 1880)**
–	Not as above	**208**
208 (207)	Distal three-winged portion of oral arms as long as proximal simple portion	***Catostylus ornatellus* (Vanhöffen, 1888)**
–	Distal three-winged portion of oral arms 2–4 times as long as proximal simple portion	**209**
209 (208)	Oral arms 2/3 the length of bell diameter	***Catostylus townsendi* Mayer, 1915**
–	Not as above	**210**
210 (209)	Oral arms 1–1,5 times the length of bell radius	***Catostylus cruciatus* (Lesson, 1830)**
–	Oral arms as long as bell diameter	***Catostylus tagi* (Haeckel, 1869)**

## Discussion

Digital resources on biodiversity can be relevant not only to researchers, but also to laypeople, such as tourists or citizen scientists. The importance of involving citizens in understanding, monitoring and protecting biodiversity has been recently expressed by the European Commission, in the document “Establishing Horizon 2020” (EU Regulation no. 1291/2013). However, most of the biodiversity-related resources available in the Web – especially the ones dedicated to to “difficult” groups, such as jellyfish – are normally devoted almost exclusively to experts (Martellos and Nimis 2015). Exposing scientific information in a form which can be accessible to everybody – without losing its content and informative value – can be a true revolution. Many citizens, especially if already interested in nature and aware of environmental issues (e.g. the presence of invasive alien species), are potentially interested in similar resources. Hence digital resources can be used to involve a wider amount of citizens in scientific tasks, such as the collection of those “big data” which are nowadays fundamental to researchers. The examples of OPAL initiative in the British Isles (http://www.opalexplorenature.org; accessed 08 August 2015) or, in the field of jellyfish, of MeteoMedusa (Boero 2013, Boero et al. 2013), and JellyWatch (http://www.jellywatch.org/; accessed 08 August 2015) are demonstrating the effectiveness of a citizen science approach in collecting scientific data.

JellyWeb is based on morpho-anatomic and taxonomic data, collected and organized in ca. 10 years of research. The development of the portal (Martellos and Nimis 2015) was based upon the experience of the European project KeyToNature (mainly devoted to digital identification) and of the project *Dryades* (devoted to the pubblication of biodiversity data in the web). This is the first portal devoted to organisms other than vascular plants developed by the research unit of the Dept. of Life Science of the University of Trieste. During its development, a particular attention was paid to user interfaces, in order to provide high quality scientific information in the most straightforward way, and to make it useable by the wider audience as possible.

The multi-entry interface can be useful to both researchers (whom can simply type the name of a taxon to retrieve related information or generate an identification key), and laypeople (whom can use it to start the identification of a jellyfish they have just seen on the seashore). As a further help, interactive keys are enriched by images and drawings of the most relevant characters. Since digital keys are generated in real time, on the basis of the list of remaining organisms, each query produces a different identification key.

Since identification is nowadays often based on molecular analysis, the system has been developed to host molecular data as well. In fact, several attempts to revise the taxonomy of the various taxa like the *Discomedusae* on the basis of morphological observations integrated with genetic analysis are underway, highlighting several critical points, such as the recognition of cryptic species in the *Aurelia* complex within the “traditional” species *Aurelia aurita* (Dawson and Jacobs 2001, Dawson and Martin 2001, Dawson 2003, Dawson et al. 2005, Ramšak et al. 2012), or even at higher taxonomic levels like the proposition of at least two new families within the *Semaeostomeae* (Bayha and Dawson 2010, Straehler-Pohl et al. 2011). The integration of molecular information in a digital identification system by using the FRIDA software was studied by Bruni et al. (2012) for vascular plants.

## Conclusion

JellyWeb is an accumulative system, which can potentially host all data on Scyphozoa, Cubozoa and Staurozoa, and even extend its aim to other groups of the phylum Cnidaria. However, a research group alone can hardly complete such a challenging task. The research unit at the University of Trieste plans to maintain and enrich JellyWeb, but its growth could be faster, if other research groups join this effort. A researcher, or a research group, can contribute to the system by:

- *Fostering a taxon* (such as a genus, or a family). This can be done by managing an instance of the FRIDA system. FRIDA allows to different authors to independently manage separate instances, while at the same time contributing to the same database of morphological ans anatomical data, hence, generating updated multi-authored keys to any subset of taxa in the whole system (for a complete description see Martellos 2010). All the digital keys which are generated by the system give credit to the authors of all the data. The keys and all the data and images in JellyWeb are always distributed under a Creative Commons share alike, by attribution 3.0 license (CC 3.0 by-sa).
- *Contributing to the image archive*. High quality images of morphological and anatomical characters and of the whole organisms are probably the most relevant bottlenecks in the process of creating a portal such as JellyWeb. Especially when identifing a taxon, digital images are of capital relevance, both for choosing among the leads of each choice, and as visual census when an identification has been achieved. Several species of Scyphozoa, Cubozoa and Staurozoa are known for one or few specimens, and, even when the taxa are well known, high quality images are, however, scarce. JellyWeb was developed to host a virtually unlimited number of images for each taxon. Each image is displayed with credits to the author(s) and owner(s), institution(s), other metadata, and license.
- *Producing descriptions*. Another relevant bottleneck in developing digital identification keys and portals to one or more groups of organisms are their descriptions. While taxonomic descriptions can be found in books and papers, descriptions which could be actually useful to people other than researches are difficult to produce. In our experience, to be appreciated by a wider audience, they should mix different sources of information, from ecology to taxonomy, from distribution to human uses, relevance for economy, etymology of the name, etc. Hence, their production is not a simple cut and paste, but a relevant effort of analysis and synthesis.

Potential contributor can contact Massimo Avian (avian@units.it), to define the extent of their participation.
